# CHRONIC IFNΓ-DRIVEN INFLAMMATION IMPAIRS INTESTINAL TOLERANCE AND REGENERATION IN AGING

**DOI:** 10.1093/geroni/igad104.0865

**Published:** 2023-12-21

**Authors:** David Castillo-Azofeifa

**Affiliations:** Genentech, Inc., South San Francisco, California, United States

## Abstract

The intestine’s remarkable regenerative capacity decreases with aging as the stem cellniche interaction is impaired. Beyond defects in the stem cell-niche interaction, there is a direct correlation between age-related changes in immunity and increased susceptibility to infections. However, how the aging immune system affects the regenerative capacity of the intestine to challenges such as infections is unclear. Here, we infected young and aged mice with Citrobacter rodentium, a model that activates the immune response and causes epithelial damage. Young mice successfully healed, while old mice succumbed to infection. In the aged infected intestines, we identified elevated basal levels of interferon γ (IFNγ) that significantly increased in lymphoid cells, CD4+, and CD8+ T cells. Additionally, aged post-infected intestines exhibited dramatic epithelial barrier disruption, partly due to a decrease in Lgr5+ stem cells and an imbalance of cell differentiation towards goblet cell production, raising the question if the aging intestine is more susceptible to infection due to persistent exposure to IFNγ. To test the response of young and aged intestinal epithelium to continuous IFNγ exposure, we treated young and aged organoids with IFNγ. We found that old organoids undergo massive apoptotic cell death as early as 24 hours post-IFNγ treatment. In contrast, young organoids were unaffected, demonstrating that IFNγ drives the age-related impairment of intestinal healing. Although a short-term boost of IFNγ is necessary for intestinal regeneration in adults, our findings indicate that prolonged IFNγ activation associated with aging can impede intestinal repair.

